# Bile Acid Signaling in Neurodegenerative and Neurological Disorders

**DOI:** 10.3390/ijms21175982

**Published:** 2020-08-20

**Authors:** Stephanie M. Grant, Sharon DeMorrow

**Affiliations:** 1 Division of Pharmacology and Toxicology, College of Pharmacy, The University of Texas at Austin, Austin, TX 78712, USA; stephanie.grant@austin.utexas.edu; 2 Department of Internal Medicine, Dell Medical School, The University of Texas at Austin, Austin, TX 78712, USA; 3 Research Division, Central Texas Veterans Healthcare System, Austin, TX 78712, USA

**Keywords:** bile acid receptors, neuroprotective, tauroursodeoxycholic acid, ursodeoxycholic acid, alzheimer’s disease, parkinson’s disease, multiple sclerosis, hepatic encephalopathy

## Abstract

Bile acids are commonly known as digestive agents for lipids. The mechanisms of bile acids in the gastrointestinal track during normal physiological conditions as well as hepatic and cholestatic diseases have been well studied. Bile acids additionally serve as ligands for signaling molecules such as nuclear receptor Farnesoid X receptor and membrane-bound receptors, Takeda G-protein-coupled bile acid receptor and sphingosine-1-phosphate receptor 2. Recent studies have shown that bile acid signaling may also have a prevalent role in the central nervous system. Some bile acids, such as tauroursodeoxycholic acid and ursodeoxycholic acid, have shown neuroprotective potential in experimental animal models and clinical studies of many neurological conditions. Alterations in bile acid metabolism have been discovered as potential biomarkers for prognosis tools as well as the expression of various bile acid receptors in multiple neurological ailments. This review explores the findings of recent studies highlighting bile acid-mediated therapies and bile acid-mediated signaling and the roles they play in neurodegenerative and neurological diseases.

## 1. Introduction

Bile acids are amphipathic molecules synthesized in the liver, stored in the gallbladder and released into the intestinal lumen in response to food intake as a digestion mechanism. Their primary function is to serve as detergents in the solubilization of dietary lipids and fat-soluble vitamins. The majority of bile acids are passively or actively recovered throughout the intestinal tract and then returned to the liver for recycling via enterohepatic circulation with a small percentage of bile acids excreted as waste. Bile acids maintain secondary functions as steroid hormones and influence metabolic processes as potent signaling molecules via membrane-bound receptors such as sphingosine-1-phosphate receptor 2 (S1PR2) and Takeda G-protein-coupled bile acid receptor 5 (TGR5) and nuclear receptors such as Farnesoid X receptor (FXR) [1]. Surprisingly, emerging evidence suggests that bile acid signaling may also play a role in the physiology and pathophysiology of the brain. Furthermore, the usage of the bile acids ursodeoxycholic acid (UDCA) and tauroursodeoxycholic acid (TUDCA) may possess therapeutic benefits in neurological ailments due to their neuroprotective properties, lack of cytotoxicity and permeability across the blood brain barrier (BBB), shown with UDCA in clinical studies [2] and TUDCA in animal models [3]. For some neurodegenerative diseases clinical trials implementing bile acid treatments may offer therapeutic potential, from a phrase III trial with UDCA [4] and to follow-up tracking of long term chenodeoxycholic acid (CDCA) efficacy [5]. Furthermore, in neurological disorders, there have been an increased amount of published studies implementing the use of bile acids or specifically targeting bile acid signaling. In this review, we focused on the signaling pathways of bile acids relevant to the CNS and their direct influence in the pathologies of neurological and neurodegenerative diseases.

## 2. Bile Acids Synthesis, Metabolism and Enterohepatic Circulation

Each day roughly 500mg of cholesterol is converted into bile acids in the adult human liver. There are two major bile acid synthetic pathways: the classic (or neutral) pathway that occurs in the liver and the alternate (or acidic) pathway found in peripheral tissues and the liver. A pathway for cholesterol regulation in the brain, the neural cholesterol clearance pathway, was discovered more recently and will be discussed at length below. In humans, cholic acid (CA) and CDCA are the only primary bile acids synthesized [6]. For rodents, their bile acid pool composition consists of primary bile acids CA, CDCA and the creation of α-muricholic acid (MCA) and β-muricholic (β-MCA) acid from CDCA [7]. Studies have reported sex differences in bile acid metabolism in healthy humans, with men displaying a higher percentage of fasting plasma concentrations of individual bile acids and total bile acids, increased by 111% and 51%, respectively [8]. Serum comparisons for bile acid profiles even showed significantly lower amounts of primary bile acids CA and CDCA when comparing women to men [9]. The variance of these findings with circulating bile acids may be useful when therapeutic drugs are being implemented for clinical trials.

**Figure 1:**
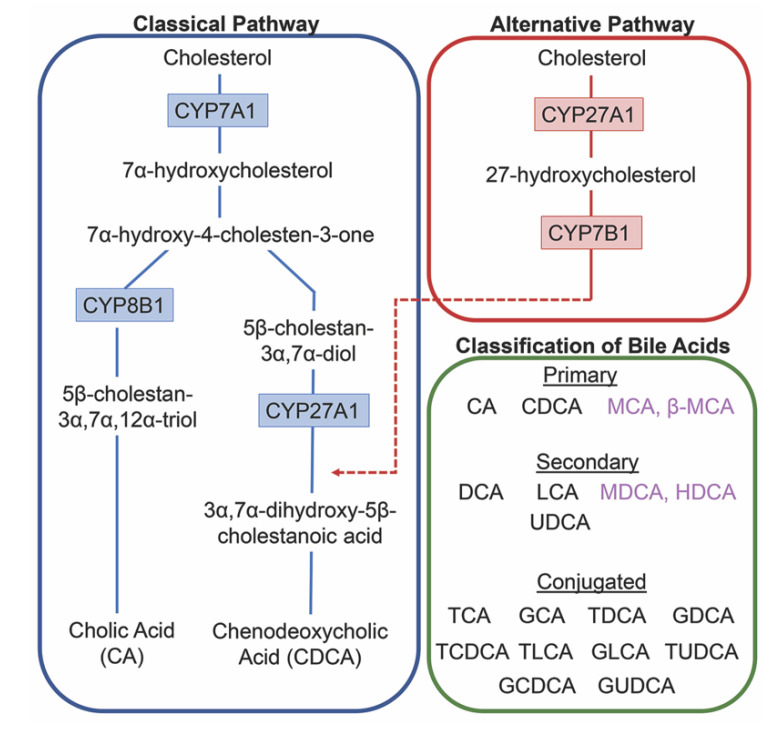
Bile acid synthesis pathways. The classic pathway for bile acid synthesis occurs in the hepatocytes of the liver via 7α-hydroxylase (CYP7A1) converting cholesterol into 7α-hydroxycholesterol. Primary bile acid cholic acid (CA) is formed after subsequent conversions from sterol 12α-hydroxylase (CYP8B1) and chenodeoxycholic acid from sterol 27-hydroxylase (CYP27A1). In the alternative or acidic pathway, mitochondrial CYP27A1 in peripheral tissues convert cholesterol into 27-hydroxycholesterol. Oxysterol 7α-hydroxylase (CYP7B1) is an additional assisting enzyme in this pathway and the resulting products feed back into the liver, indicated by the red arrow feeding into the classical pathway under CYP27A1. Primary and secondary bile acids specific to rodents are listed in purple. Bile acids can become conjugated with glycine or taurine after interactions with gut flora.

The conversion of cholesterol to primary bile acids is facilitated by a family of unique cytochrome P450 enzymes that are located in the cytosol, endoplasmic reticulum, mitochondria, and peroxisomes. Expressed solely in the hepatocytes, the classic bile acid synthesis pathway is initiated via 7α-hydroxylase (CYP7A1) converting cholesterol into 7α-hydroxycholesterol with the resulting metabolic products of primary bile acids synthesized to CA via sterol 12α-hydroxylase (CYP8B1) or CDCA by sterol 27-hydroxylase (CYP27A1) [10]. In contrast, the alternative pathway catabolizes cholesterol in all tissues; cholesterol is metabolized via mitochondrial CYP27A1, converting it into 27-hydroxycholesterol. For further conversion, these midpoint metabolites are transported from peripheral tissues back to the liver to be converted to primary bile acids CA and CDCA [11]. The classic pathway is the primary route for bile acid synthesis regulated by CYP7A1, the only rate-limited enzyme in all bile acid synthesis. More than 90% of total bile acid production in humans is sourced from this pathway, with less than 10% of the total bile acids coming from the alternative pathway during routine physiological conditions [7]. In contrast, in healthy wild type mice, only 60% of their total bile composition is sourced from the classical pathway due to their bile acid pool, including the addition of MCA and β-MCA that are not present in healthy humans [12]. Enterohepatic circulation allows the total bile salt pool to undergo 4–12 cycles a day, an efficient method of reabsorption and recycling that ensures minimal bile acid loss via urinary or fecal excretion [13]. A summary of the bile acid synthesis pathways can be seen in Figure 1.

Before de novo primary bile acids CA and CDCA are released from the liver, some are conjugated with either glycine (in humans) or taurine (in mice), granting increased water solubility and reduced cytotoxicity to fulfill their dietary roles of lipid emulsification throughout the intestines. Taurocholic acid (TCA) and glycocholic acid (GCA) are synthesized from CA, taurochenodeoxycholic acid (TCDCA) and glycochenodeoxycholic acid (GCDCA) are synthesized from CDCA. Along with CA and CDCA, these newly synthesized conjugated bile acids are transported from hepatocytes to the bile canaliculus via the bile salt export pump (BSEP) and multidrug resistance-associated protein 2 (MRP2) for storage in the gallbladder awaiting their release into the intestinal lumen with the intake of food. Once nutrients enter the stomach, they trigger the gallbladder to release bile acids into the duodenum where they contribute to the digestion of lipids and fat-soluble vitamins. As bile acids continue through to the ileum, unconjugated and some glycine-conjugated bile acids will be reabsorbed via passive diffusion in the jejunum and colon with the majority of conjugated bile acids requiring active reabsorption via the apical sodium dependent bile acid transporter (ASBT) in the ileum. Other active membrane transporters sodium taurocholate cotransporting polypeptide (NTCP) and organic anion transport polypeptide (OATP) in hepatocytes mediate in bile acid reuptake once they’ve entered portal venous circulation [14].

**Figure 2:**
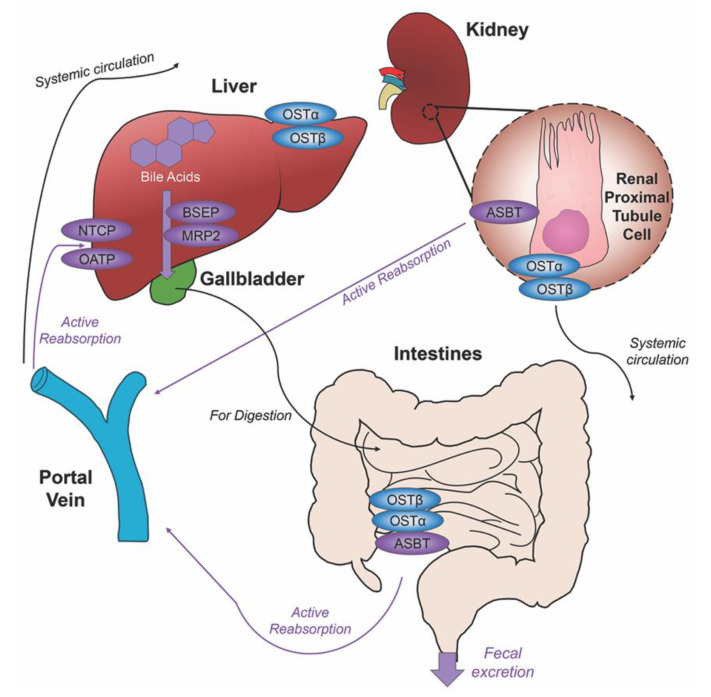
Enterohepatic circulation of bile acids. After primary bile acids are synthesized in the liver, the bile acid transporters bile salt export pump (BSEP) and multidrug resistance-associated protein 2 (MRP2) facilitate their storage in the gallbladder, indicated via thick purple arrow, to be released in the intestines to aid in the digestion of food. Following food intake, bile acids are released into the duodenum for the digestion of lipids and fat-soluble vitamins, bile acid movement indicated via black arrow. Some bile acids can be reabsorbed through passive diffusion in the jejunum and colon throughout the journey, while the majority of conjugated bile acids can interact with the apical sodium dependent bile acid transporter (ASBT) in the ileum for active reabsorption, indicated by multiple purple arrows. Other bile acid transporters sodium taurocholate cotransporting polypeptide (NTCP) and organic anion transport polypeptide (OATP) expressed in hepatocytes mediate active reabsorption back to the liver. Bile acids in systemic circulation will be reabsorbed by ASBT in the renal proximal tubule cells of the kidney and directed back to the liver via the portal vein. Heteromeric organic solute transporter (OST) α and β in renal proximal tubule cells, ileocytes and hepatocytes direct bile acids into systemic circulation. The efficiency of this system recycles and minimizes fecal and urinary bile acid loss by excretion.

Unconjugated primary bile acids not passively reabsorbed will interact with the intestinal bacteria flora present in the colon, creating secondary bile acids deoxycholic acid (DCA) from CA and lithocholic acid (LCA) and UDCA from CDCA for humans [15], with the secondary bile acids of murideoxycholic acid (MDCA) and hyodeoxycholic acid (HDCA) for mice [16]. When these secondary bile acids are recirculated back to the liver, conjugation with glycine or taurine can further differentiate them, such as the addition of taurine to UDCA forms TUDCA [17]. The enterohepatic circulation efficiently reclaims approximately 95% of bile acids and minimizes fecal and urinary bile acid expulsion with the help of a collective transporter process. Located on the membranes of ileocytes, proximal renal tubule cells and cholangiocytes, ASBT facilitates the absorption of the majority of bile acids lacking passive diffusion qualifications or reclaims bile acids in systemic circulation for portal venous distribution back to the liver, minimizing excretion in urine. The heteromeric organic solute transporter (OST) α and β located in the cytosol of renal proximal tubule cells, ileocytes and hepatocytes direct bile acids to systemic circulation [18], a process which when malfunctioning could exacerbate elevated systemic bile acids levels affecting the blood brain barrier [19,20]. A graphic depiction of the enterohepatic circulation of bile acids is shown in Figure 2.

Bile acids can undergo an additional elimination pathway consisting of glucuronidation, a process that converts hydrophobic bile acids into excretable metabolites. Uridine 5′-diphosphate-glucuronosyltransferase (UDP-glucuronosyltransferase, UGT) are multigenic enzymes that catalyze the glucuronidation reaction, conjugating glucuronic acid with exogenous and endogenous molecules. Aiding in bile acid detoxification, the resultant hydrophilic glucuronide products possess increased ability for urinary excrement [21]. Glucuronidation of bile acids leads to the important introduction of a negative charge to the molecule, allowing transport by conjugate-transporters that can facilitate bile acid-glucuronide secretion. Multidrug resistance-associated protein 1 (MRP1) and 3 (MRP3) expressed across the basolateral hepatocyte membrane aid in the efflux of glucuronides [22]. Bile acid glucuronides are present in hepatic dysfunction, with increased concentrations of glucuronidated bile acids CDCA and LCA in the plasma of patients with hepatobiliary diseases [23]. In biliary obstruction patients whose bile flow had been restored via stenting, the urinary composition of bile acid glucuronides was increased [24].

Several UGT genes have been identified in human, mouse, rat and other mammalian species. The gene superfamily consists of four UGT families, *UGT1*, *UGT2*, *UGT3* and *UGT8*, with enzymes of *UGT1* and *UGT2* families the most efficient at glucuronic acid transfer [25]. Of the 18 UGT enzymes, three enzymes can be attributed to glucuronidation of bile acids: UGT2B4 for bile acids such as HDCA, UGT2B7 for primary, secondary and hydroxylated bile acids, and lastly UGT1A3 for bile acids such as CDCA, LCA, and HDCA [26]. While many UGT isoforms are predominately expressed in the liver, these enzymes are also expressed in a variety of extrahepatic tissues including the small intestines, colon, bladder, kidney, ovaries, uterus, testis, and stomach [27]. UGT expression levels and glucuronidation activity have been detected in all nine regions of the rat brain [28] and are present in the human brain [29]. Lastly, UGT has been identified in several neural cell types: neurons [30], astrocytes [30,31] and microglia [32].

## 3. Bile Acids in the Brain

**Figure 3:**
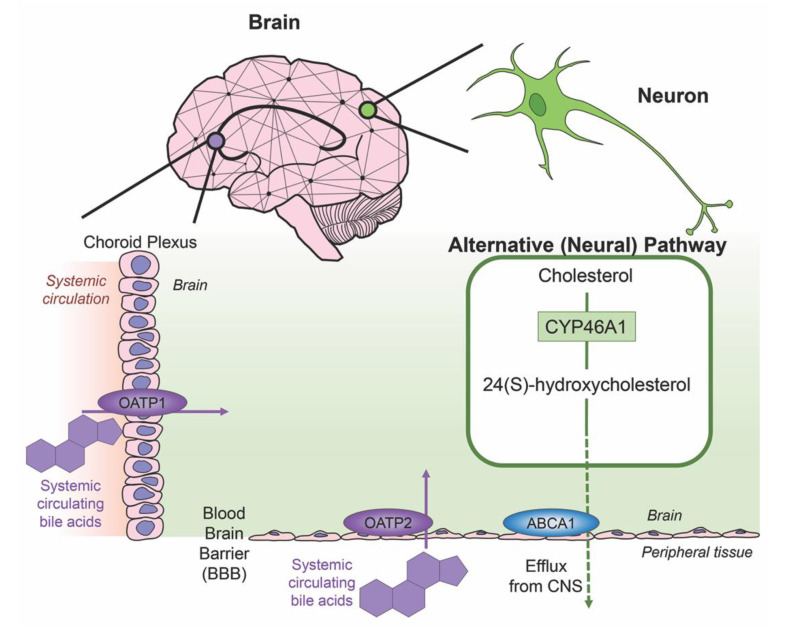
Neural cholesterol clearance pathway and bile acid transport into the CNS. Cholesterol is catalyzed in the brain via sterol 24-hydroxylase (CYP46A1), an enzyme expressed only in neurons. It is converted to 24(S)-hydroxycholesterol and is able to be removed from the CNS through the blood brain barrier (BBB) via the transporter ATP-binding cassette transporter 1 (ABCA1), indicated via green arrow. Other transporters mediate systemic circulating bile acids into the CNS. Organic anion transporter polypeptide 1 (OATP1) expressed in the choroid plexus and organic anion transporter polypeptide 2 (OATP2) expressed at the BBB both mediate the transport of bile acids, both processes indicated by purple arrows.

Cholesterol is an essential component of neural development and in the composition of neurons and neuroglia, with nearly 25% of the total body cholesterol found in the brain [33]. It is a major component of the lipid molecules in the membranes of neuron and glial cells, a large fraction in the myelination performed by oligodendrocytes and is involved in the synthesis of steroid hormones [34]. While local cholesterol biosynthesis is observed at higher rates in glial cells than neurons [35], neurons solely possess the ability for cholesterol clearance. The last alternative pathway for bile acid synthesis is in the brain (shown in Figure 3), catalyzing cholesterol by neuron-specific sterol 24-hydroxylase (CYP46A1) and converting this into 24(S)-hydroxycholesterol; the increased solubility of this intermediate allows for efflux from neural tissue via the BBB through lipoprotein transport ATP-binding cassette transporter 1 (ABCA1) [36]. Specific bile acid transporters allowing the influx of bile acids from the periphery into the CNS also exist. For example, OATPs, with rat OATP1 expressed in the choroid plexus and rat OATP2 highly expressed at the BBB allow for the influx of bile salts and a variety of other amphipathic organic compounds into the CNS [37]. Similarly, subpopulations of neurons, particularly in the hypothalamus express the transported ASBT which facilitates the internalization of bile acids into neurons where they have been shown to influence the activity of the hypothalamic–pituitary–adrenal (HPA) axis [38,39].

Bile acid functionality increases when acting as ligands for nuclear receptors farnesoid X receptor (FXR), the pregnane X receptor (PXR), the vitamin D receptor (VDR), and membrane receptors Takeda G-protein-coupled receptor 5 (TGR5; a G-protein-coupled receptor also called G-protein-coupled bile acid receptor 1, GPBAR-1), sphingosine-1-phosphate receptor 2 (S1PR2) and α5β1 integrin. These receptors are highly expressed in the liver and the intestines but also display activity in a variety of tissues throughout the body including the brain [40]. Bile acids can act as signaling molecules to modulate their own homeostasis. Among the bile acid receptors, FXR plays many important roles in the regulation mechanisms of bile acid synthesis and transport. FXR activation via bile acids can induce the expression of BSEP [41,42], regulating the canalicular secretion of bile acids into bile. Key players in bile acid synthesis, CYP7A1 and CYP27A1, can be repressed by FXR [22] and human UGT2B4, involved in the conversion of hydrophobic bile acids to their less toxic glucuronide derivatives, can be upregulated by FXR [43].

The affinities of primary, secondary and conjugated bile acids with individual bile acid receptors vary. Bile acids can serve as weak activators of the glucocorticoid receptor (GR) in the brain to influence the HPA axis [39]. LCA, a hydrophobic and cytotoxic bile acid, has been shown as a weak ligand for FXR with the ability to decrease BSEP expression [44]. This same bile acid is the most potent bile acid for TGR5 [45], displaying anti-tumor effects in human neuroblastoma cell cultures [46] as well as pro-apoptotic effects in breast cancer cells [47]. The primary bile acid CDCA is the most potent activator for FXR, with CA and secondary bile acids DCA and LCA showing less activation [48]. Both nuclear receptors PXR [49] and VDR [50] can be activated by secondary bile acid LCA. There are even conjugated bile acids with selective activity for receptors, such as TUDCA for α_5_β_1_ integrin [51] and TCA for S1P2R [52]. Other reviews have eloquently covered the liver and intestinal focused signaling of these receptors [11,53,54,55]. Given these points, Table 1 lists bile acid-mediated receptors that are relevant in the CNS.

## 4. Bile Acids in Neurodegenerative Diseases

**Figure 4:**
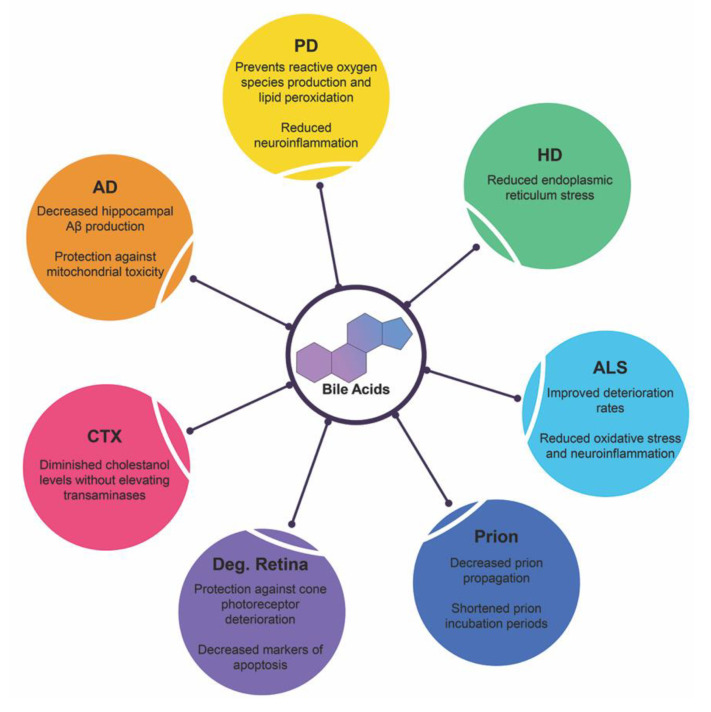
Neuroprotective functions of bile acids in neurodegenerative diseases. Recent clinical trials and experimental animal studies have shown the protective qualities of therapeutic bile acids in these disease states. Abbreviations: Alzheimer’s disease (AD), Parkinson’s disease (PD), Huntington’s disease (HD), amyotrophic lateral sclerosis (ALS), prion diseases (Prion), degenerative retina diseases (Deg. Retina), Cerebrotendinous xanthomatosis (CTX).

Affecting millions worldwide, neurodegenerative diseases stem from a variety of factors. The exact mechanism underlying the route of pathogenesis for each disease state varies but commonality exists between them all: accumulation of misfolded/mutated protein and aberrant pathways of endoplasmic reticulum stress leading to increased dysfunction, widespread neuronal loss and cerebral atrophy. Below is recent research highlighting bile acid signaling and its therapeutic potentials in these debilitating diseases and is summarized in Figure 4.

### 4.1. Alzheimer’s Disease

The most common cause of dementia, Alzheimer’s disease (AD) is an aggressive and fatal degenerative disease with etiologies stemming from the combination of aggregated beta-amyloid plaques and tau tangles, neuroinflammation, massive neuronal demise, and cerebral atrophy [73]. The burden of this disease is magnified by the progressive decline in cognitive and motor functions, marked by subjective cognitive decline (the worsening memory loss and inability to remember common routine tasks), confusion with time/location and distinct changes in mood and personality [74]. Different length Aβ are produced by the amyloid precursor protein (APP). The terminal which results from subsequent proteolytic cleavages yields various Aβs, with the Aβ42 peptide as the most linked to disease development due to hydrophobicity and liability of aggregation. Animal models utilized to understand AD pathogenesis focus on mutations of human genes *APP*, *PSEN1* and *PSEN2* that modulate amyloid β peptides (Aβ) via the γ-secretase complex and lipid metabolism via apolipoprotein E (ApoE) which focuses on Aβ clearance mechanisms [75].

The presence of bile acids in AD is an emerging topic of research, with altered bile acid compositions providing novel insight. A recent clinical study took plasma from 30 healthy controls, 20 subjects with mild cognitive impairment and 30 subjects with clinical AD and performed widespread bile acid testing. Levels of LCA were significantly increased in AD patients compared to the controls whereas levels of glycochenodeoxycholic acid, glycodeoxycholic acid and glycolithocholic acid were significantly elevated in AD patients compared to mild cognitive impairment patients. The presence of LCA and these glycine-conjugated bile acids demonstrate helpful biomarker qualities for diagnostic purposes [76], although how these bile acids may be contributing to the pathogenesis of AD is unknown.

In pre-clinical studies, targeting bile acid signaling has also been identified as a potential experimental therapy to alleviate various aspects of AD. Using a surgical model of AD, Aβ toxicity induced via single intracerebral ventricular injection of Aβ_1-42,_ treatment with INT-777 (6α-ethyl-23(S)-methylcholic acid, a TGR5 agonist) significantly attenuated the cognitive impairment and decreased neuroinflammation, as measured by decreased proinflammatory tumor necrosis factor-α (TNF-α), interleukin-1β (IL-1β), and interleukin-6 (IL-6) cytokine production and microglia activation [77]. In contrast, an in vitro study using an SH-SY5Y treated Aβ_1-42_ AD cell model noted FXR overexpression triggered neuronal apoptosis via activation of the cAMP-response element-binding protein (CREB)/brain-derived neurotrophic factor (BDNF) pathway [78]. Furthermore, the addition of 6α-ethyl-chenodeoxycholic acid (6ECDCA), an FXR agonist, aggravated the Aβ-induced apoptosis, whereas knockdown of FXR in these cells, both basal and Aβ-induced, inhibited neuronal apoptosis [78]. Together these data would suggest opposing actions of the bile acid receptors TGR5 and FXR in the pathogenesis of AD.

Other studies utilized bile acids to alter different mechanisms of AD pathogenesis, from impeding Aβ production to improving mitochondrial function. A rat model of AD neurotoxicity using intraperitoneal injections of AlCl_3_ for six weeks noted that daily injections of CDCA significantly attenuated AlCl_3_-induced cognitive and spatial deficits markedly similar to the control and decreased hippocampal Aβ production via Aβ42 levels. Hematoxylin and eosin staining morphologically indicate a CDCA neuroprotective effect on the control and CDCA + AlCl_3_ groups when compared to the severe neuronal degradation in the AlCl_3_-treated group [79]. Lastly, mitochondrial damage and morphological abnormalities are implicated in patients with sporadic and familial AD and factors such as dynamin-related protein 1 (Drp1), are known to protect against AD-related mitochondrial toxicities [80,81]. Treatment with the bile acid UDCA exerts a neuroprotective effect on mitochondrial membrane potential and morphology of primary fibroblasts through fission and fusion modulator Drp1 [82]. Taken together, while data to suggest that bile acids and bile acid signaling is involved in the pathogenesis of AD are sparse, the use of bile acids as therapeutic options for the treatment of AD is promising.

### 4.2. Parkinson’s Disease

After AD, Parkinson’s disease (PD) is the second most common neurodegenerative disease marked by progressive motor deterioration. Dopaminergic neuronal death and α-synuclein-containing Lewy bodies in the substantia nigra are two known characteristics although the majority of PD cases are of sporadic origin [83]. Animal models replicate this pathology using neurotoxins, genetic mutations or combinations of the two [84]. Phenotypically, clinical diagnosis of PD is more recognizable at later stages, when motor deficits are apparent due to the misfolded α-synuclein proteins spreading to additional parts of the brain and subsequently affecting the substantia nigra. However therapeutic options starting prior to the onset of motor symptoms (prodromal phase), would be the most beneficial in slowing the disease progression thus highlighting the importance of identifying key biomarkers for successful diagnosis [85].

PD research utilizing surgical rodent models of PD observed bile acid metabolism alterations and potential bile acid markers. Using a prodromal PD mouse model created by injecting human α-synuclein fibrils and human α-synuclein monomers (as a control) via stereotactic unilateral injection, serum and brain tissue from the mice was analyzed for metabolomics. Metabolite pathway analysis in the brain tissue of the α-synuclein fibrils treated mice yielded significant alterations of four biochemical pathways: taurine and hypotaurine metabolism, bile acid biosynthesis, glycine, serine and threonine metabolism and the citric acid cycle. The taurine and hypotaurine metabolism pathway that was disrupted includes taurine which has the crucial role in the conjugation of neuroprotective TUDCA and UDCA [86]. An adeno-associated virus-α-synuclein injected bilaterally into the substantia nigra of rats noted that overexpression of α-synuclein, which additionally is expressed in enteric neurons, altered their gut microbiome. Along with diversifying the gut microbiome, this overexpression significantly increased the level of free bile acids and primary bile acids (CA, total MCA and β-MCA), and additionally increased secondary bile acids (taurodeoxycholic acid, taurohyodeoxycholic acid and DCA) irrespective of influence from exercise [87]. Another study further clarified the presence of bile acids using a surgical mouse prodromal PD model, with three being found significantly decreased in the serum of the α-synuclein-fibrils-treated group: omega-muricholic acid, TUDCA and UDCA. UDCA and TUDCA, both neuroprotective secondary bile acids that can pass the BBB, were markedly affected with a 17- and 14-fold decrease from the control group [88]. These surgical rodent model studies, replicating aspects of PD, shows increased research in this field will assist in therapeutic changes.

Other recent PD research has used anti-inflammatory secondary bile acids TUDCA and UDCA in experimental therapy studies. Decreased mitochondrial activity has been implicated in PD; the mitochondrial inhibitor 1-methyl-4-phenyl-1,2,3,6-tetrahydropyridine (MPTP) replicating glial activation and the pro-inflammatory cytokine cascade of PD. A series of TUDCA injections were introduced prior to and after the MPTP-injection in a mouse model of PD. Motor capabilities improved in the MPTP-treated + TUDCA groups in comparison to MPTP-treated mice along with the ability to initiate movements and amend tremors. Parkin levels, an E3 ubiquitin ligase associated with mitochondrial biogenesis, were decreased in MPTP-treated mice and were attenuated in mice treated with TUDCA prior to MPTP [89]. This same group looked into dopaminergic cell death, oxidative stress and reactive oxygen species (ROS), using the same MPTP-induced PD mouse model. SH-SY5Y cells were treated with 1-methyl-4-phenylpyridinium (MPP^+^) or doxycycline for two in vitro PD models, displaying TUDCA’s antioxidant qualities in both by preventing ROS production and lipid peroxidation through increased nuclear factor erythroid 2 related factor 2 (Nrf2) expression. TUDCA’s neuroprotective potential was replicated in vivo with the MPTP-induced mouse model, reverting ROS production caused by MPTP and increasing the expression of Nrf2 and Nrf2 downstream cytoprotective enzymes, glutathione peroxidase and heme oxygenase-1 [90]. Lastly, a rotenone-induced PD model using rats with daily intraperitoneal injections of UDCA resolved striatal dopamine content close to the control group level and significantly downregulated nuclear factor-κB (NF-κB), BCL2 associated X apoptosis regulator (Bax) and caspase-9 mRNA levels. Striatal TNF-α and IL-1β levels that were significantly increased in the rotenone-treated group were attenuated in the UDCA administered group. Additionally, this UDCA treatment reduced rotenone-induced alterations of striatal neuron mitochondrial and increased striatal ATP to 2-fold above the control values [91]. PD research implementing bile acid-mediated therapeutics has attenuated several harmful cellular mechanisms of this disease state.

### 4.3. Huntington’s Disease

Huntington’s disease (HD) is an inherited autosomal-dominant neurodegenerative disease classified by progressive motor degeneration, cognitive disorder and neuropsychiatric decline. The mutant gene huntingtin, *HTT*, on chromosome four induces neuronal loss in the striatum and causes multiple irregularities such as cellular proteostasis, mitochondrial and synaptic dysfunction through mutant 7–35 cytosine-adenine-guanine (CAG) repeats. The end product of multiple CAG repeats lengthens glutamine residues on the mutant huntingtin protein leading to accumulation and toxicity [92].

Clinical and rodent-focused animal research in HD lacked consideration of the direct effects of bile acid signaling but rather focused on noteworthy alterations found in pathways related to the enzymes in bile acid synthesis. One study noted a link between brain cholesterol homeostasis and a reduction of CYP46A1, the enzyme initiating cholesterol clearance in neural tissue, levels in the striatum of post-mortem patients of HD, transgenic R6/2 mice (a rodent HD model) and a striatal neuron progenitor line expressing mutant *HTT*. Gene therapy using a stereotaxic injection of adeno-associated virus (AAV)rh10 viral constructs for GFP or human CYP46A1 restored CYP46A1 levels in striatal neurons of R6/2 mice and increasing neuronal survival through production of sterols laneosterol and desmosterol, metabolites of CYP46A1 processing, and reestablishment of normal cholesterol levels [93]. Sphingosine-1-phosphate metabolism in HD patients and two rodent models has shown aberrant signaling of intermediates and metabolizing enzymes, with increased expression of sphingosine-1-phosphate lyase and decreased expression of sphingosine kinase 1/2 in the striatum of post-mortem humans and HD transgenic models, both in early and late stages of the HD rodent model [94]. Decreasing the bioavailability of sphingosine-1-phosphate could dismantle downstream signaling from G-protein coupled receptors sphingospine-1-phosphate receptors 1–5 [95] of which S1PR2 has been shown to have expression in neurons [62].

Other HD research delved into alternative experimental methods for creative solutions to HD’s characteristic protein accumulation. Aggregation of mutant huntingtin protein, the trademark of the disease, and the association of ER stress mechanisms has shown causative pathogenesis conditions in HD [96]. One study observed low molecular weight chemical chaperones to reduce protein accumulation and misfolding due to their ability to pass through the BBB. TUDCA showed an initial significant reduction in thapsigargin-induced ER stress comparable to other chaperones of the study (4-phenylbutyrate and docasahexaenoic acid), but the dosage response had diminished efficacy even after higher concentrations were administered [97]. Extending beyond solely implementing bile acids therapeutics, this recent HD research has shown that looking at indirect changes involving pathways related to bile acid synthesis pathways can be progressive prognosis tools.

### 4.4. Amyotrophic Lateral Sclerosis

Amyotrophic lateral sclerosis (ALS) is a motor neuron disease marked by deterioration of the upper and lower motor neurons of the brain stem and spinal cord resulting in muscular atrophy, paralysis and a patient survival prognosis of 2–5 years. Mutations of chromosome 9 open reading frame 72 (*C9orf72*), fused in sarcoma (*FUS*), superoxide dismutase *(SOD)1*, and transactive response DNA-binding protein 43 (*TARDBP/TDP-43*) genes are commonly associated with ALS pathogenesis [98]. Animal models include glial cells along with neurons among affected cell types, with ER stress, autophagy and RNA metabolism dysfunction. Rodent ALS models are primarily transgenic knockouts of SOD1 and TDP-43 variants, with SOD1 mutant mice the only rodent model with a phenotype similar to ALS in humans [99].

The efficacy of targeting bile acid signaling as a therapeutic strategy for the treatment of ALS has been assessed in a Phase II clinical trial. A double-blind placebo controlled clinical trial was performed with a 54-week TUDCA daily oral treatment in 34 ALS patients currently taking riluzole as an add-on regimen. The treatment was well tolerated in all patients without any severe adverse effects beyond common gastrointestinal symptoms. TUDCA treatment for 1 year has potential neuroprotective effects with slowed deterioration of function in ALS patients, with a 15% increase in ALS functional rating scale (ALSFRS-R) scoring [100]. Due to the aggressive nature of ALS, studies that improve deterioration rates or increase neuronal growth show a promising future for ALS research.

Other recent ALS research focused on experimental studies with bile acid therapies instead of targeting specific bile acid signaling. An in vitro ALS model using motor neuron-like NSC-34 cells expressing wild type or G93A mutation of human SOD1 and treated with glycoursodeoxycholic acid (GUDCA). Treatment with GUDCA diminished caspase-9 levels and the amount of apoptotic nuclei present, regardless of treatment occurring at the beginning or after cell differentiation of NSC-34 cells transfected with mutated G93A. Oxidative stress and neuroinflammatory mediators of nitric oxide production and metalloproteinase-9 (MMP-9) were attenuated by GUDCA therapy, but extracellular ATP levels remained depleted [101]. Another study combined both in vitro and in vivo experiments. Human G93A mutated motor neuron cultures determined that, amongst several others, prior treatment of bile acids TCA, TUDCA and taurine-glycine-conjugated cholic acid 45 min prior to cyclopiazonic acid (CPA) addition, a mycotoxin that inhibits calcium ATPase in the ER and selectively targets motor neurons over other cell types to induce ER stress, rescued 50% of neurite growth. TUDCA displayed strong neurite outgrowth-promoting effects but insignificant motor neuron survival or relevant ER stress-related gene expression. In a smaller study with mice expressing mutated G93A, human SOD1 were used for early disease state ALS during a presymptomatic period in the hind limb muscle. Subcutaneous injections of TUDCA every 3 days for 21 days yielded a significant increase in neuromuscular junction innervation when compared to vehicle-treated animals, attenuating one of the earliest phenotypes observed in ALS mouse models [102]. With the maximum patient survival prognosis of 5 years due to the rapid deterioration of this disease, this bile acid-centric research promoting neurogenesis is a step in the right direction.

### 4.5. Prion Diseases

Structured around the deviant aggregation of membrane-bound prion protein PrP^C^ found on human gene *PRNP*, prion diseases are incurable neurodegenerative diseases derived from sporadic (Creutzfeldt–Jakob disease, CJD), genetic (familial CJD, fatal familial insomnia and Gerstmann-Straussler-Scheinker disease) or acquired (kuru and iatrogenic CJD) origin. The pathogenic conformation PrP^Sc^, composed of approximately 47% β-sheet in relation its benign counterpart, induces neurotoxicity and a variety of rapid manifestations of neuronal degeneration [103]. Due to the highly protease-resistant and seeding properties of PrP^Sc^, research targeting rapid detection is crucial for therapeutic manipulations to address primary and secondary nucleation. Various rodent models of prion diseases aid in therapeutic progression with accurate expressions of the disease phenotype; direct intracerebral inoculation of PRNP or mutated PRNP transgenic mice lines have all been produced [104]. Mechanisms inducing the phosphorylation of eukaryotic translation initiation factor, eIF2α, are linked to ER stress, unfolded protein response (UPR) activation and neurodegeneration [105].

Novel prion disease research conducted has implemented experimental bile acid treatment to strictly target and delay protein aggregation plaguing prion diseases. One study looked at a series of anti-prion compounds and their effects of formation kinetics with TUDCA among them. TUDCA treatment resulted in a delay in prion fibril formation and blocked seeding in a shaking-induced conversion model for prion conversion. However, additional analysis for time-dependent prion oligomer and fibril formation yielded no anti-prion effects [106]. Another aggregation study of different mouse prion strains (RML, 22L and ME7) with TUDCA produced inhibition of lag phases and prevented exponential growth when compared to groups without TUDCA implementation. Nontoxic treatments of TUDCA and UDCA in RML-infected mouse neuroblastoma cells diminished preliminary PrP^Sc^ levels after the second passage but neither fully cleared proteinase resistant prions even after six passages. The neuroprotective elements of TUDCA and UDCA against prion propagation could additionally be observed in prion-infected cerebellar slice cultures: treatment with either bile acid at day 14 maintained levels of granule and Purkinje cells for 49 days when compared to RML-treated slices; starting treatment at 21 days post-infection yielded less beneficial effects [107]. 

Other studies utilized rodent prion disease models and implemented TUDCA treatments to explore dysfunctional cellular mechanisms attributed to the disease state. Another recent study compared different secondary bile acid-mediated therapies and the effects on ER stress, a relevant cellular mechanism of the disease state. A gender-difference rodent model of prion disease implemented treatment trials of TUDCA and UDCA and generated several results. Treatment of 0.4% TUDCA in chow 7 days post inoculation increased incubation periods and significantly increased phosphorylated eIF2α levels in TUDCA-treated infected male mice when compared to control infected male mice. Increased dosage to 1% TUDCA with the same experimental manipulations yielded no statistical difference in incubation periods or levels of prion protein, aggregation, ER stress markers (binding immunoglobulin protein (BiP), phosphorylated-eIF2α) or neuronal loss (neuronal nuclei, NeuN)/synaptic activity postsynaptic density protein 95, PSD95). Interestingly, treatment with 1% UDCA 100 days post inoculation, to replicate later-stage disease relevance, significantly shortened the incubation period in both mice genders yet provided a diminished survival effect—levels of PSD95 and BiP were significantly increased in UDCA-treated infected mice when compared to untreated infected female mice. Further immunohistological examinations of mice with shorter survival rates displayed symptoms consistent with prion disease and not due to UDCA toxicity [108]. While prion disease remains a fatal affliction, the increase in research implementing bile acid therapy to delaying and subsequently diminishing prion-specific protein accumulation will prove fruitful to understanding this disease state.

### 4.6. Degenerative Retina Diseases

Retinal degeneration diseases involve the deterioration, dysfunction and death of light-sensitive neurons, called photoreceptor cells, that leads to incurable blindness. Layered retinal cytoarchitecture combines distinguished layers of rod and cone bipolar (outer plexiform layer), amacrine (inner nuclear layer) and ganglion (inner plexiform layer) cells that establish and transfer synaptic information through to the brain via the optic nerve [109]. Combinations of genetic mutations (i.e., mutant variants of genes Peripherin or retinal pigment epithelium (RPE)65), morphological changes of the retinal pigment epithelium (RPE) and photoreceptor dysfunction (i.e., photoreceptor-specific transcription factor CRX) contribute to multiple degenerative retina diseases: glaucoma, retinitis pigmentosa (RP), age-related macular degeneration (AMD) and inherited retinal degeneration (RD). Commonly used animal models consist of zebrafish for ocular development studies, primates and other large animals with macula for human disease comprehension, and multiple transgenic mice strains with retinal and photoreceptor degeneration and cone-rod dystrophy phenotypes [110].

Recent research has focused on rodent models of experimental bile acid therapies to study the origins of photoreceptor degeneration in retina diseases. One study highlights TUDCA ability to interact with rhodopsin via a spectroscopic assay measuring the stability of rhodopsin’s photoactivated form, metarhodopsin II. Three different confirmation models of TUDCA computer docking to the binding site on metarhodopsin II gave plausible options due to the energy minimum [111]. An RP transgenic rodent line, the rd1 mouse, detected the effect of daily TUDCA intraperitoneal injections on morphological photoreceptor deterioration. TUDCA provided protective effects to cone photoreceptor function at P21 and preserved the outer nuclear layer and significant quantities of photoreceptor nuclei in the retinas of TUDCA-treated mice when compared to vehicle-treated mice [112]. Another study observed the apoptotic and oxidative stress hallmarks in RD’s photoreceptor degeneration via a chemically-induced model from administration of N-methyl-N-nitrosourea with subcutaneous treatments of TUDCA. Along with preserving retinal thickness and cone photoreceptors, expressions of apoptotic markers Caspase-3, Calpain-2 and Bax were significantly downregulated when compared to RD-induced mice. TUDCA additionally alleviated oxidative stress and increased expression of endogenous antioxidant superoxide dismutase (SOD) in TUDCA-treated mice [113].

Other studies observed degenerative retina diseases in rodent models to monitor gene expression and the effects of various bile acid-mediated treatments. An retinitis pigmentosa GTPase regulator (RPGR) conditional knockout mouse model of RP with weekly TUDCA treatments was utilized to determine signaling-transduction mechanisms of rhodopsin. Mutations of RPGR are the common cause of RP and the RPGR protein complex a regulator of protein trafficking in the retina. RPGR knockout mice displayed varied locations of rhodopsin, opsin and transducing when compared to wild type mice. Expression of nephrocystin-4 (NPHP4), a component of the RPGR unit, was also absent in connecting cilium along with scaffold protein NOD-like receptor family pyrin domain containing receptor 3 (NLRP3) colocalization with microglial neuroinflammation marker ionized calcium binding adaptor molecule 1 (IBA1) in early stages of RPGR knockout mice morphology, a potential contributor to degeneration. Treatment with TUDCA significantly attenuated photoreceptor loss when compared to untreated knockout mice and significantly reduced microglia activation, mimicking morphological similarities to the wild type group [114]. Lastly, an in vitro model of AMD using retinal pigment epithelial and choroid endothelial cells; TCA treatment maintained tight junction structure and function affected by AMD-induced oxidative stress and inhibited choroidal angiogenesis [115]. Despite the varied mechanisms and disease states, bile acid therapies have shown protective qualities in multiple aspects of degenerative retina diseases.

### 4.7. Cerebrotendinous Xanthomatosis

Cerebrotendinous xanthomatosis (CTX) is an autosomal recessive lipid storage disorder caused by mutations in the CYP27A1 gene creating dysfunctional bile acid metabolism, afflicting patients with a progressive disarray of symptoms including but not limited to: ataxia, dementia, epilepsy, tendon xanthomas and cataracts. The mutated gene leads to a reduction in the formation of CDCA and an upregulation of cholesterol 7α-hydroxylase, elevating levels of 7α-hydroxy-4-cholesten-3-one and subsequently serum and urine levels of cholestanol and bile alcohol. Administration of bile acids for replacement therapy improves the symptoms CTX patients face, with CDCA the predominant choice for treating both neurological and non-neurological symptoms [116]. CYP27A1 transgenic mice do not form xanthomas in the tendons or the brain, with the level of accumulated cholestanol not replicating that of CTX patients. These mice are useful for mechanistic evaluations of CTX but still not an ideal animal model for the disease [117].

Clinical studies for CTX are abundant in this field, targeting alternative therapies for reconstructing the aberrant bile acid synthesis pathways or identifying potential disease biomarkers. One study highlighted the efficacy of using CA, instead of the standard CDCA, with significantly diminished cholestanol levels without elevated transaminases. The adverse effect of altered transaminases and subsequently cholestasis has been shown in the traditional CDCA treatment in a neonatal CTX case [118]. Another group differentiated indirect bile acid synthesis biomarkers in an extensive long-term study, with significant elevation of plasma cholestanol and serum lathosterol in untreated patients. However, notably, serum 27-hydroxycholesterol (a bile acid metabolite) was very low or absent in all CTX patients despite CDCA treatment, highlighting its efficacy as an overall prognosis marker at any stage in the disease [119]. Different methods of bile acid replacement therapy and identifying possible significant metabolites will aid in improving CTX patients’ quality of life.

## 5. Neurological Disorders and Bile Acids

The presence of bile acids continues to have increased relevance in maladies of the CNS beyond neurodegenerative diseases. With aspects of neuroinflammation, neural cell injury and overall deterioration playing a role in these disease states, studies implementing therapeutic techniques or targeting bile acid signaling have shown neuroprotective value. Current research highlights these findings and the mechanisms behind them.

### 5.1. Multiple Sclerosis

Multiple sclerosis (MS) is a chronic inflammatory disease of the CNS characterized by demyelinating processes, immune-mediated neuroinflammation and axonal dysfunction leading to motor, sensory and cognitive deterioration. There are several phenotypes: relapsing-remitting MS (RRMS), primary progressive MS (PMS), and secondary progressive MS (SPMS) [120]. Due to the symptoms of MS varying by the area of the CNS that is affect, there are several animal models utilized to study clinical manifestations: experimental autoimmune encephalomyelitis, viral-induced encephalomyelitis, or chemical/toxin-induced demyelination [121].

Identification of bile acid synthesis pathway intermediates, metabolites and bile acid receptors in MS has been shown in clinical studies. Several metabolomic analyses yielded differences in bile acid metabolism of patients afflicted with MS when compared to controls. One study indicates a significant increase in (25R)26-hydroxycholesterol, a precursor in the alternative pathway in bile acid synthesis, in the plasma of patients with RRMS [122]. Another showed significant increases in the ratio of DCA metabolites to CA metabolites in the plasma of patients with PMS. Furthermore, the increased expression of receptors FXR and TGR5 were identified in positive-stained white matter lesions of PMS autopsy tissue. Using the experimental autoimmune encephalomyelitis mouse model, TUDCA treatment reduced the severity of demyelination and astrocytosis through the effects of TGR5 signaling [123].

Other studies have focused on the expression of FXR in MS and the downstream effects of FXR signaling. One group noted the expression of FXR was downregulated in MS patients, more so in PPMS than RRMS patients and an in vivo treatment with FXR agonist GW4064 yielded significant diminished inflammatory markers when compared to vehicle-treated mice. Myeloid cell-mediated FXR activation increased levels of anti-inflammatory IL-10; making FXR a novel regulator for autoimmunity and mediating CNS inflammation of MS [124]. Another study looked into FXR activity in autoimmunity of MS via orally active synthetic FXR agonist obeticholic acid. Toxin-induced MS in FXR knockout mice treated with the synthetic agonist diminishes active and passive MS pathology more than CDCA treatment, but significantly increased proinflammatory IL-6. FXR agonists possess a causative role in influencing T-cell and B-cell expression as well as markers of apoptosis in activated T-cells [125]. Taken all together, the increased presence of bile acid metabolites and moderating FXR expression for MS therapies will increase our knowledge of this disease state.

### 5.2. Hepatic Encephalopathy

Hepatic encephalopathy (HE) is a neuropsychiatric disorder that creates neurological, cognitive and functional deterioration caused by multifactorial elements of three routes of liver impairment: acute liver failure (type A HE), portal-systemic shunting (type B HE) and cirrhotic liver damage (type C HE). Clinical stages of HE progress from minimal HE (some alterations without clear mental change), covert HE (mild abnormalities of awareness, cognitive abilities and sleep fluctuations) to overt HE (ataxia, asterixis, confusion and bizarre behavior and progression to coma) [126]. Associated with the cognitive deficits is hyperammonemia and the presence of neuroinflammation that are thought to co-ordinately contribute to the pathogenesis of HE [127]. Several well-characterized animal models for HE exist depending on each disease phenotype, toxin-induced or via surgical means, for the varied aspects of HE pathogenesis [128].

Studies have shown fluctuations of bile acid levels, underlying the significance of BBB permeability in this disease state and how this could affect neurological dysfunctions. Metabolomic analysis of 14 patients with overt HE displayed increased bile acid levels when compared to the levels of controls. Cerebrospinal fluid levels of bile acids possessed a 93-fold and 241-fold increase for GUDCA and GCA, respectively, highlighting BBB permeability in HE [129]. Furthermore, serum analysis of cirrhotic patients with and without HE, displaying a significant increase in total and conjugated bile acids as well as levels of GCA, GCDCA, TCA and TCDCA. Using animal models of Type A HE, increased total bile acid content in brain tissue has been demonstrated [19], and is associated with an increase in the bile acid TCA specifically [62], although the individual species of other bile acids contributing to this tissue specific increase is unknown.

The involvement of aberrant bile acid signaling in the pathogenesis of HE has been demonstrated in preclinical studies. Strategies to reduce the increased serum bile acids that occur during liver damage, either by feeding mice the bile acid sequestrant cholestyramine [19], using Cyp7A1 knockout mice with a reduced bile acid pool [19], or inhibiting ASBT activity in the intestine to prevent bile acid reabsorption into the blood stream [20], attenuated the cognitive deficits observed in a various models of Type A HE. Furthermore, it has been demonstrated that the increase in serum bile acid during liver damage/failure may be contributing to the hyperpermeability of the blood brain barrier [130] that is observed during HE.

Other HE studies specifically target bile acid signaling and their contributions to HE pathogenesis with in vitro and rodent HE models, the majority of which focus on FXR signaling. Specifically, in a model of Type A HE, FXR expression has been shown to increase in neurons of the frontal cortex [19] and that intracranial infusion of an FXR-specific vivo morpholino to knockdown the expression of FXR attenuated the cognitive deficits observed in this HE model [19], suggesting that aberrant FXR signaling maybe contributing to the pathogenesis of HE. It is thought that the consequences of aberrant neuronal FXR activation during HE may be a dysregulation of cholesterol homeostasis [58]. Specifically, a decrease in the expression of Cyp46A1, a component of the neural cholesterol clearance pathway, was observed which resulted in a concomitant increase in cholesterol content in the brains of mice with Type A HE [58]. Strategies to inhibit FXR receptor activity attenuated the dysregulation of cholesterol homeostasis in the brain during HE [58]. Neural bile acid signaling during HE also has implications for the neuroinflammatory processes and signals. For example, an in vitro neuroinflammation model using LPS and primary microglia showed decreased proinflammatory cytokines IL-1β and IL-6 when co-treated with taurolithocholic acid (TLCA) [131]. Furthermore, S1PR2 expression can be seen in neurons, directly increasing the expression of proinflammatory cytokine chemokine ligand 2 (CCL2) upon interacting with bile acid TCA [62]. CCL2, has been shown as a key regulatory chemokine regulating the activation of microglia during HE [132]. Conversely, TGR5, expressed in neurons and microglia, was found to be upregulated in the cortex of a type A rodent HE model, whereas activating this receptor suppressed CCL2 neuronal paracrine signaling and reduced microglial-induced inflammation [60]. Taken together, these studies highlight the involvement of bile acid signaling in the pathogenesis of HE and may prove an effective therapeutic target for the development of strategies to manage this complex disease.

### 5.3. Miscellaneous Neurological Disorders

Other neuropathies induced by physical damage or genetic mutations also have been studied through experimental studies involving bile acid-mediated therapy.

Traumatic brain injury (TBI) is a heterogenous trauma-based disorder caused by physical injury to the head. Axonal and neuronal cell injury, neuroinflammation and neurodegeneration responses in acute, subacute and chronic TBI stages are potential protein biomarkers for disease tracking [133]. One study looked into therapeutic roles of TUDCA in mechanism of TBI’s disease state. The ratio of protein kinase B (Akt)/p-Akt was shown to be decreased in a rodent model of TBI along with the expression of 78-kDa glucose-regulated protein (GRP78), an indicator of ER stress. The neuroprotective role of TUDCA solely influenced the Akt/p-Akt pathway by decreasing ER stress and improved disease conditions by decreasing neuronal apoptosis, improving secondary brain injury factors of TBI [134]. Another group displayed significant bile acid signaling in a TBI animal model. A significant decrease in ASBT-expressing neurons was observed in the hypothalamus of a rodent model of TBI, which could adversely contribute to inflammatory responses [135].

X-linked adrenoleukodystrophy (XALD) is a neurometabolic disorder caused by mutations in the *ABCD1* gene that leads to progressive axonopathy of ascending and descending sensory and motor spinal cord tracts. Mitochondrial dysfunction, accumulated very long chain fatty acids, cerebral inflammation and demyelination contribute to the pathophysiology of the disease [136]. Clinical studies showing markers of irregular bile acid metabolism have given useful XALD insight. Metabolic screening of XALD patients found significant decreases in plasma bile acid levels, with unconjugated CA and CDCA showing the most significant reduction. Lathosterol, a marker of cholesterol synthesis reflective of bile acid malabsorption, was significantly reduced as well when compared to control subjects [137]. Another study focused experimental therapy to look into ER stress, an XALD disease mechanism. Diet supplementation of TUDCA in genetic mouse models of XALD reduced UPR activation (significantly increasing levels of GRP94, GRP78 and significantly decreasing protein disulphide isomerase (PDI) levels) and attenuated axonal degeneration (significantly decreasing markers of synaptophysin and APP in spinal cord axons) [138].

While TBI and XALD vary greatly in terms of the origin of their pathology, both have been included in studies with experimental methods driven by neuroprotective bile acids and targeting ER stress mechanisms that affect both disease states.

## 6. Conclusions

In summary, the therapeutic implementation of bile acids and targeting bile acid-mediated signaling in neurodegenerative and neurological diseases cannot be overstated. Research that includes bile acids and their specific receptors in neurology is continually expanding due to multiple factors: bile acids have neuroprotective properties that can be seen replicated in treatment regimens (both in clinical and experimental animal models), bile acid receptors have been located in the brain and specific several neural cell types, and bile acids possess the ability to permeate through the BBB thus offering simple and feasible pharmacological options. The prevalence of bile acids in neurological research is a growing field and will continue to expand beyond the scope of hepatic and cholestatic diseases, provide novel discoveries and strategies for controlling these debilitating diseases.

## Figures and Tables

**Table 1:** Bile Acid Receptors in the CNS.

Receptor	Bile Acid Ligands	Cellular Localization	Expression/Functionality	References
FXR	CDCA, CA, DCA, LCA	Cortical neurons	Nuclear and cytoplasmic expression in cortical neurons; transcriptional activity via SHP activation. FXR deletion elevates cerebellar neurotransmitter concentrations. FXR modulates cholesterol metabolism in a rodent model of type A hepatic encephalopathy.	[56,57,58]
TGR5	LCA, DCA, CDCA, CA	Neurons, astrocytes, microglia	Response to neurosteroids resulting in increased intracellular cAMP. TGR5 signaling is neuroprotective and diminishes inflammation against CCL2 in a rodent model of type A hepatic encephalopathy	[59,60]
S1P2R	TCA, GCA, TDCA, GDCA, TUDCA	Cortical neurons, microglia, hippocampal pyramidal cells, retinal ganglion cells	Mediates synaptic neuroplasticity, repair and neurite outgrowth. TCA activation promotes inflammation in a type A rodent model of hepatic encephalopathy	[61,62]
PXR	LCA	Brain endothelial cells, hippocampal neurons	BBB regulation via ABC-transporters, nonyphenol toxicity activates PXR-mediated apoptosis and neurotoxicity	[63,64]
VDR	LCA	Neurons, glia	Location of VDR indicates involvement with neurosteroids, confirmation of nuclear location	[65,66]
α_5_β_1_ integrin	TUDCA, norUDCA (UDCA homolog)	Cortical neurons, brain endothelial cells	Regulates neural morphology and migration during development, α5 influence BBB permeability	[67,68,69]
GR	UDCA, TCA, GCDCA, TUDCA	Neurons, microglia, cortical neurons	UDCA-bound GR modulates NF-κB-dependent transcription, GR-signaling in ginseng has protective implications in neurodegenerative models, GR-mediated HPA axis suppression is induced via injection of bile acids, GR attenuates amyloid-beta-induced apoptosis in cortical neurons through TUDCA	[39,70,71,72]

## Abbreviations

6ECDCA	6α-ethyl-chenodeoxycholic acid
AAV	Associated virus
Aβ	Amyloid β peptides
ABCA1	ATP-binding cassette transporter 1
AD	Alzheimer’s Disease
Akt	Protein kinase B
ALS	Amyotrophic Lateral Sclerosis
AMD	Age-related macular degeneration
ApoE	Apolipoprotein E
APP	Amyloid precursor protein
ASBT	Apical sodium-dependent bile acid transporter
Bax	BCL2 associated X apoptosis regulator
BBB	Blood brain barrier
BDNF	Brain-derived neurotrophic factor
BiP	Binding immunoglobulin protein
BSEP	Bile salt export pump
C9orf72	Chromosome 9 open reading frame 72
CA	Cholic acid
CAG	Cytosine-adenine-guanine
CDCA	Chenodeoxycholic acid
CJD	Creutzfeldt-Jakob disease
CCL2	Chemokine ligand 2
CPA	Cyclopiazonic acid
CREB	cAMP-response element-binding protein
CTX	Cerebrotendinous Xanthomatosis
CYP	Cytochrome P450
DCA	Deoxycholic acid
Drp1	Dynamin-related protein 1
eIF2α	Eukaryotic translation initiation factor
FUS	Fused in sarcoma
FXR	Farnesoid X receptor
GCA	Glycocholic acid
GCDCA	Glycochenodeoxycholic acid
GR	Glucocorticoid receptor
GRP78	78-kDa glucose-regulated protein
GUDCA	Glycoursodeoxycholic acid
HD	Huntington’s disease
HDCA	Hyodeoxycholic acid
HE	Hepatic Encephalopathy
HPA	Hypothalamic pituitary adrenal
IBA1	Ionized calcium binding adaptor molecule 1
IL-1β	Interleukin-1β
IL-6	Interleukin-6
LCA	Lithocholic acid
MCA	α-Muricholic acid
β-MCA	β-Muricholic acid
MDCA	Murideoxycholic acid
MMP-9	Metalloproteinase-9
MPP^+^	1-methyl-4-phenylpyridinium
MPTP	1-methyl-4-phenyl-1,2,3,6-tetrahydropyridine
MRP1	Multidrug resistance-associated protein 1
MRP2	Multidrug resistance-associated protein 2
MRP3	Multidrug resistance-associated protein 3
MS	Multiple Sclerosis
NeuN	Neuronal nuclei
NF-κB	Nuclear factor-κB
NLRP3	NOD-like receptor family pyrin domain containing receptor 3
NPHP4	Nephrocystin-4
Nrf2	Nuclear factor erythroid 2 related factor 2
NTCP	Sodium taurocholate cotransporting polypeptide
OATP	Organic anion transport polypeptide
OST	Organic solute transporter
PD	Parkinson’s Disease
PDI	Protein disulphide isomerase
PMS	Primary progressive Multiple Sclerosis
PSD95	Postsynaptic density protein 95
PXR	Pregnane X receptor
RD	Retinal degeneration
RRMS	Relapsing-remitting Multiple Sclerosis
ROS	Reactive oxygen species
RP	Retinitis pigmentosa
RPE	Retinal pigment epithelium
RPGR	Retinitis pigmentosa GTPase regulator
S1PR2	Sphingosine-1-phospphate receptor
SOD	Superoxide dismutase
SPMS	Secondary progressive Multiple Sclerosis
TBI	Traumatic brain injury
TCA	Taurocholic acid
TCDCA	Taurochenodeoxycholic acid
TDP-43	Transactive response DNA-binding protein 43
TGR5	Takeda G-protein-coupled bile acid receptor 5
TLCA	Taurolithocholic acid
TNF-α	Tumor necrosis factor-α
TUDCA	Tauroursodeoxycholic acid
UDCA	Ursodeoxycholic acid
UDP	Uridine 5′-disphosphate
UGT	Uridine 5′-disphoasphate-glucuronosyltransferase
UPR	Unfolded protein response
VDR	Vitamin D receptor
XALD	X-linked adrenoleukodystrophy
